# Intraoral Involvement in Linear Scleroderma En Coup De Sabre: A Case Report

**DOI:** 10.7759/cureus.55886

**Published:** 2024-03-10

**Authors:** Nuha A Alkanhal, Haifa AlKhodier

**Affiliations:** 1 Pediatric Dentistry, King Faisal Specialist Hospital and Research Centre, Riyadh, SAU

**Keywords:** oral soft tissue, dental, rare, autoimmune, linear scleroderma

## Abstract

Localized scleroderma is a connective tissue disorder that causes excessive collagen deposition and skin fibrosis. It can be subdivided into morphea and linear scleroderma. En coup de sabre (ECDS) is a rare variant of linear scleroderma typically found among children. It is usually treated with methotrexate and corticotherapy in addition to folic acid supplements. To date, few cases of ECDS have been reported with oral involvement. This case report discusses a seven-year-old girl with linear scleroderma ECDS who was referred to the dental clinic to evaluate muscular hypotrophy on the floor of the mouth. Upon clinical and radiographic examination, the patient had hypotrophied mylohyoid muscle, reduced alveolar bone height on the affected side, and a deviated midline to the affected side as well. Furthermore, the patient was classified as having a high caries risk. After consultation with the primary physician regarding treatment modalities and options, the patient completed her comprehensive dental treatment at the Dental Department at King Faisal Specialist Hospital and Research Centre in Riyadh, Saudi Arabia.

## Introduction

Scleroderma is an autoimmune disease that causes excessive collagen deposition and skin hardening. However, its manifestations can be seen in extracutaneous tissue as well [1,2]. Morphea is characterized by skin thickening and increased quantities of collagen in the lesion. It is subdivided into linear scleroderma, plaque morphea, deep morphea, bullous morphea, and generalized morphea. Moreover, the major two types of scleroderma are localized and systemic. In the localized type, the muscles and bones are affected in addition to the skin, whereas in the systemic type, more organs might be involved such as kidneys, heart, and lungs [3-6]. The localized form is more commonly reported among the pediatric population, whereas there is no difference in the prevalence of the systemic type between the adult population and the pediatric population [5]. Additionally, localized scleroderma can be distinguished from the systemic type by the absence of Raynaud’s phenomenon, microvascular changes, internal organ involvement, and autoantibodies [5]. Furthermore, localized scleroderma can be subdivided into linear scleroderma and morphea [3,7]. 

Linear scleroderma en coupe de sabre (ECDS) (progressive facial hemiatrophy) is a rare form of linear scleroderma that affects the limbs, frontoparietal face, and scalp with possible organ involvement and neurological complications with an increased incidence among pediatric patients [8-11].

The etiology of linear scleroderma is unknown; however, it is linked to possible genetic sequences that are believed to be triggered by trauma, infection, and some medications [5,12].

The diagnosis of the disease can be confirmed histopathologically [12-14]. The appearance depends on the stage of the injury [5]. At early stages, lymphocytic infiltrate can be visualized around the vessels, and collagen fibers increase in thickness. In the progressed stages of the condition, the collagen fibers get harder and the infiltrate fades. The increase in the thickness of the collagen fibers decreases the diameter of the lumen of the blood vessels [5,15].

The most commonly used medication to treat the condition is methotrexate. It can be prescribed as an oral medication or as an injectable drug either alone or in combination with cortisone [16-20]. Furthermore, an 80% success rate has been reported with the following drug regimen in particular: 1mg/kg/week to be given subcutaneously, with the maximum recommended dose being 25mg/week [19,20].

To date, few cases of linear scleroderma have been reported with oral involvement. The purpose of this case report is to expand the frame for the clinical presentation of linear scleroderma ECDS and shed light on the head and neck area.

## Case presentation

Initially, a three-year-old girl was referred by a local hospital to the Department of Rheumatology King Faisal Hospital and Research Centre in Riyadh, Saudi Arabia to evaluate facial hyperpigmentation that was associated with atrophy that started over the hair and then progressed in size and number. 

The patient was diagnosed with linear morphea (en coup de sabre). Using a non-enhanced volumetric axial computed tomography technique of the brain and orbits with sagittal and coronal reformations, it was confirmed that the patient was cleared of any intracranial or intraorbital abnormality. Furthermore, the patient did not have any neurologic symptoms, vision complications, or weakness. The patient was followed up for many years. Initially, she was treated with topical corticosteroids (minoxidil, protopic, mometasone) with no improvement. Then, the treatment regimen was changed to tocilizumab (8 mg/kg) given intravenously every four weeks combined with methotrexate (0.5mg/kg) (total of 10 mg) given orally every week. Additionally, the following supplements were given folic acid (5 mg) weekly and vitamin D (2000 IU) daily. 

At the age of seven years, the patient was referred to the Department of Dentistry to evaluate the hypotrophied mandible and the muscles under the tongue.

**Figure 1:**
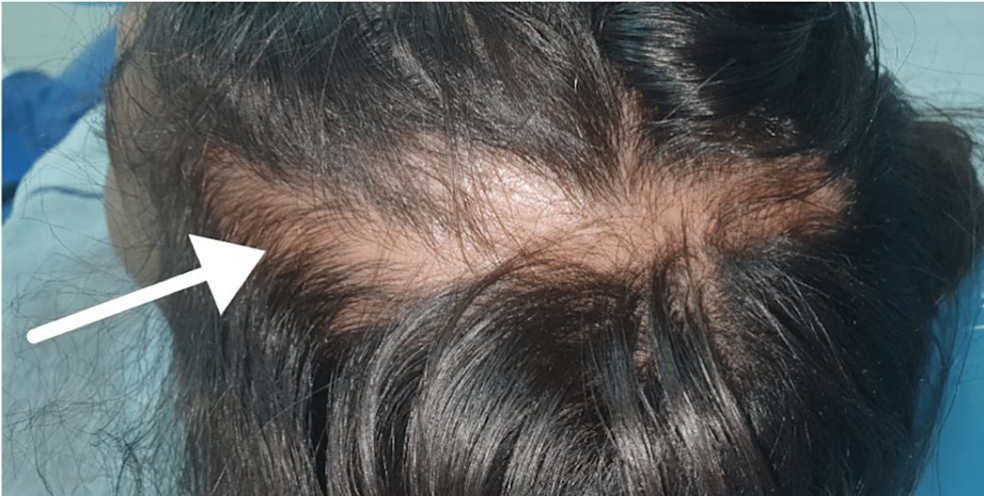
Scarring over the left frontoparietal area

**Figure 2:**
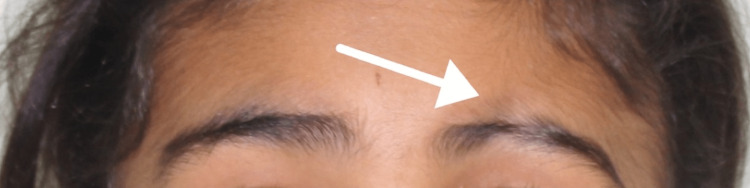
Depressed supraorbital cutaneous/subcutaneous tissue over the left eyebrow

**Figure 3:**
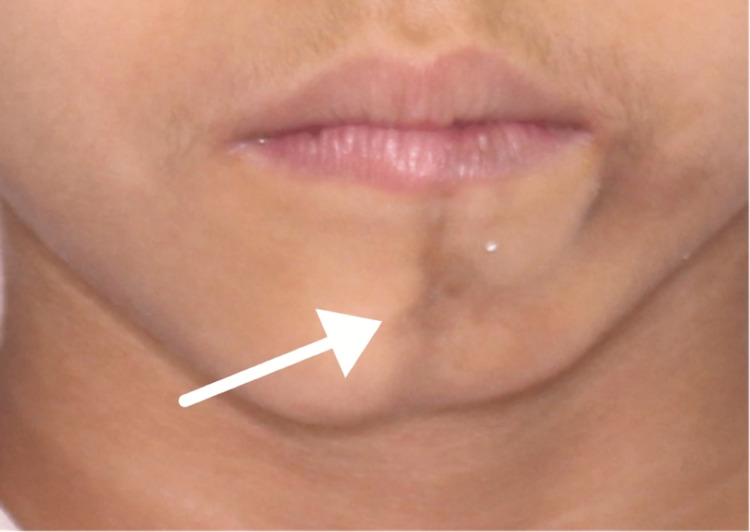
Underdeveloped left side of the mandible

Upon clinical examination, the patient presented with multiple cicatricial patches over the scalp combined with scarring over the left frontoparietal area (Figure 1), depressed supraorbital cutaneous/subcutaneous tissue (over the left eyebrow) (Figure 2), and underdeveloped left side of the mandible (Figure 3).

**Figure 4:**
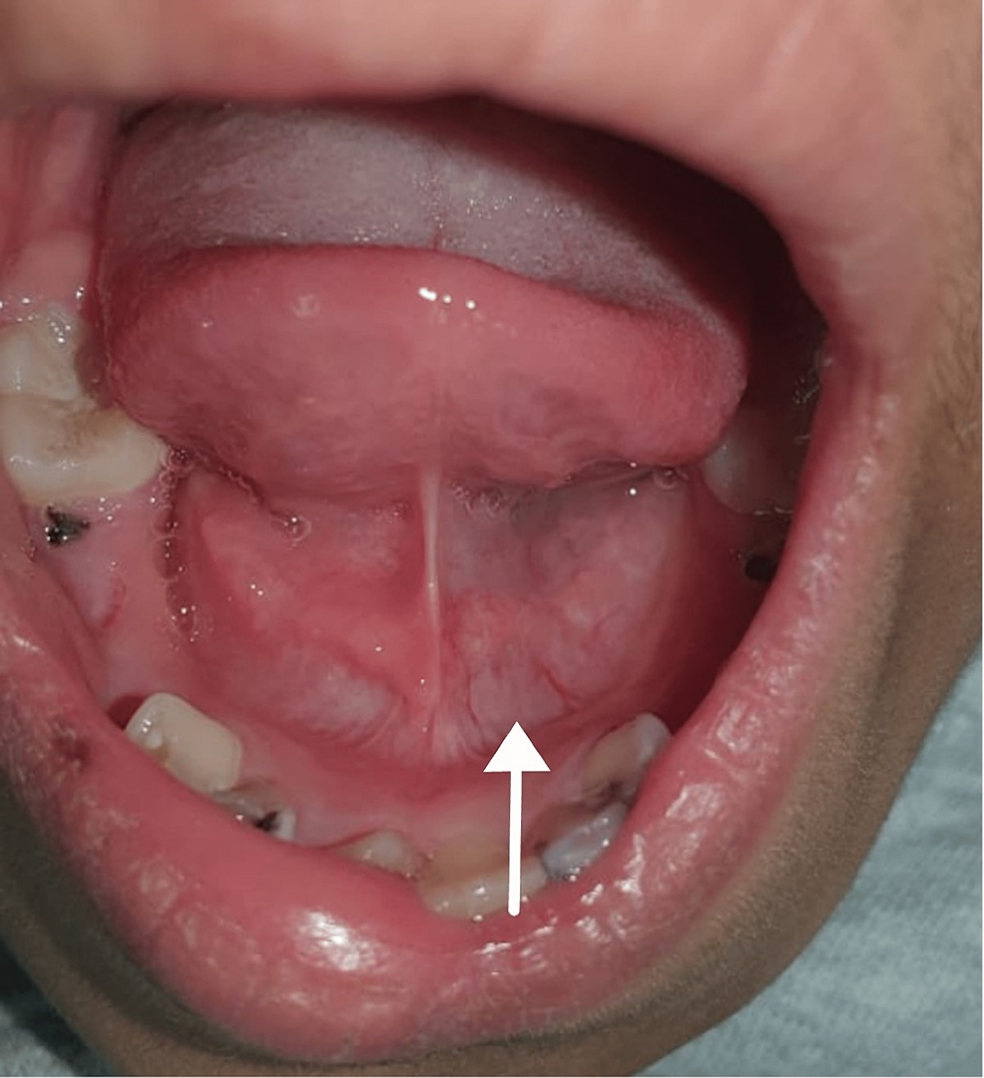
Hypotrophied left sublingual fold

**Figure 5:**
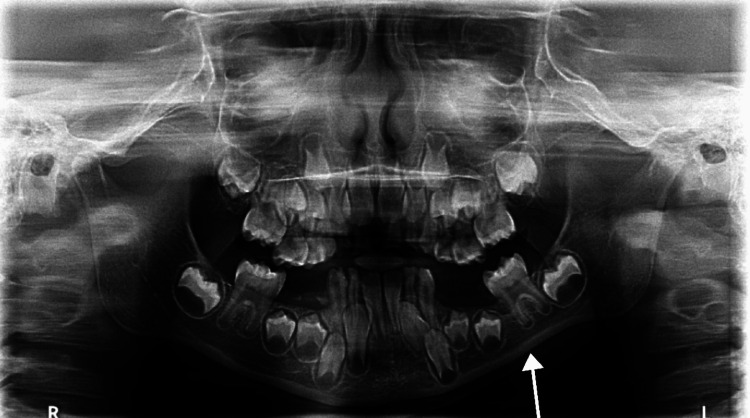
A panoramic X-ray showing a decreased alveolar bone height on the left side of the mandible

Intraorally, the patient had hypotrophied left sublingual fold (Figure 4) and decreased alveolar bone height on the left side of the mandible as shown in the panoramic X-ray (Figure 5). 

**Figure 6:**
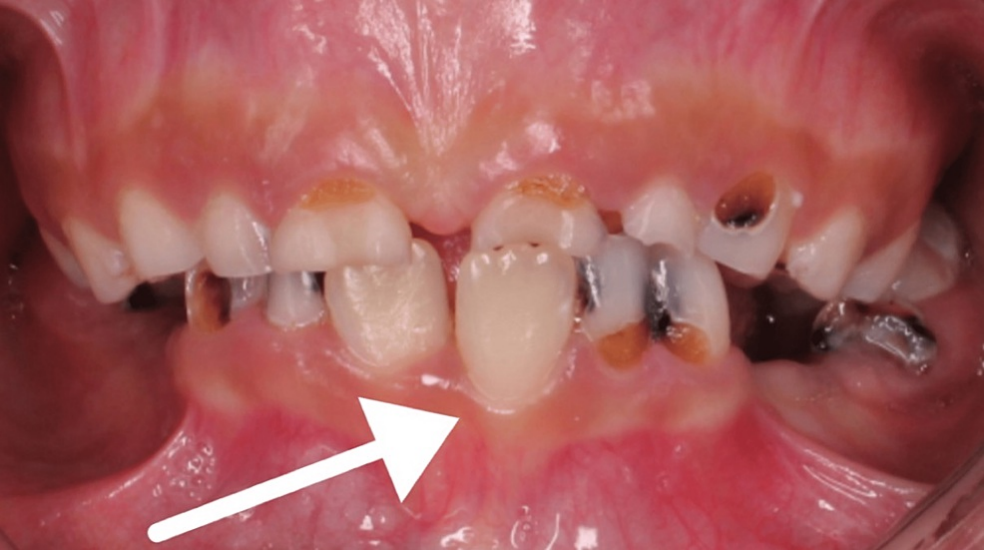
Buccally displaced #24

Additionally, the patient presented with misaligned lower anterior segment (Figure 6), poor oral hygiene, multiple carious lesions, non-compliance with oral hygiene instructions, and unfavorable dietary habits. 

After the patient completed her regular dental treatment, oral hygiene instructions were reinforced again with the sitter to avoid the development of new carious lesions, especially in the newly erupting permanent teeth, thus, preventing infection, further deterioration, and subsequent possible loss of permanent teeth. Finally, if deemed necessary in the future placing implants may require bone grafting in the left side of the mandible. At this stage, this intervention is neither required nor necessary. Currently, the patient is stable and her disease is inactive with no progression. Furthermore, the lesions are getting softer and lighter with no evidence of new lesions and currently, the patient is being followed up for periodic recalls and topical fluoride applications.

## Discussion

Scleroderma is an autoimmune connective tissue disease that can be subdivided into systemic or localized. Systemic scleroderma or systemic sclerosis is characterized by skin fibrosis and visceral organ involvement such as the lungs, kidneys, and blood vessels, whereas localized scleroderma is characterized by excessive collagen deposition and inflammation that affects the skin and the underlying tissue [6]. Localized scleroderma can be divided into two major categories: linear (band-like lesion) and morphea (plaque lesions) [3,7]. Linear scleroderma is more common among children and in some scenarios not limited to the skin and can progress to the systemic type involving multiple internal organs [5,6]. It is more common among females when compared to males with a ratio of 3:1 with 13 years of age being the average time of onset. Moreover, it was found that the duration of skin lesions’ activity is 2-5 years [5].

Neurological and ophthalmological complications are commonly reported among the pediatric population [1,8]. In our case, the patient was a seven-year-old female with a similar diagnosis whose computed tomography of the brain and orbits (sagittal and coronal reformations) cleared her from any intracranial or intraorbital involvement.

The treatment regimen for this condition varied among physicians with methotrexate being reported as the most commonly used one as a solo medication and in some situations in conjunction with corticotherapy [16-20]. The patient in our report received 8 mg/kg tocilizumab intravenously every month combined with 0.5mg/kg methotrexate orally given every week. Additionally, the following supplements were given: 5 mg of folic acid every week and 2000 IU of vitamin D daily.

Linear scleroderma ECDS progresses at a slow rate and usually affects one side of the face. However, it can be bilateral [3]. In the current case report, the patient had a unilateral pattern affecting the left side of the face and the head.

In general, an area of tissue contraction will form a depression on the frontoparietal face and scalp. Additionally, the affected area on the scalp will have linear alopecia [3]. Moreover, the depression may affect the nose, bones of the skull, upper lip, tongue, and underlying alveolus. Additionally, the teeth may be spaced and misaligned or may have delayed eruption [12-14]. In this case, the patient presented with linear alopecia over the left parietal area combined with multiple cicatricial patches over the scalp. She had a depressed left eyebrow, an underdeveloped chin on the left side, an underdeveloped left sublingual fold in addition to a buccally displaced #24 (lower left central incisor). Furthermore, panoramic radiography findings showed an underdeveloped mandibular alveolar ridge on the left side which could affect any future plan for implant placement if deemed necessary for any reason. 

A number of cases were presented with similar clinical and radiographical findings as ours. However, the extension and aggressiveness varied according to the progression of the disease at the time these cases were reported. Extensive dental displacement can lead to dental malocclusion, attachment loss, and possible difficulty in maintaining good oral hygiene, especially among children. Furthermore, severe bone involvement can lead to skeletal malocclusion that requires both orthodontic and maxillofacial surgery intervention. This case adds to the extension of scope for the clinical presentation of linear scleroderma ECDS. Further literature review of similar case reports is encouraged to assess the incidence of oral findings among this population of patients.

## Conclusions

Linear scleroderma ECDS is a rare connective tissue disease that occurs more commonly among the pediatric population. It affects the skin, muscles, and underlying bone structure of one side of the face. However, it could be bilateral. Although it is not commonly reported, it has intracranial and ophthalmological manifestations. From a dental point of view, it may result in skeletal malocclusion, reduced alveolar height, and dental malalignment which may have an impact on periodontal attachment and the oral hygiene routine.

## References

[1] Kashyape P, D'Souza AP, Fathalla B (2020). En coup de sabre presenting as status epilepticus. Clin Rheumatol.

[2] Lis-Święty A, Skrzypek-Salamon A, Ranosz-Janicka I, Brzezińska-Wcisło L (2017). Localized scleroderma: clinical and epidemiological features with emphasis on adulthood- versus childhood-onset disease differences. J Eur Acad Dermatol Venereol.

[3] Careta MF, Romiti R (2015). Localized scleroderma: clinical spectrum and therapeutic update. An Bras Dermatol.

[4] Denton CP, Khanna D (2017). Systemic sclerosis. Lancet.

[5] Fett N, Werth VP (2011). Update on morphea: part I. Epidemiology, clinical presentation, and pathogenesis. J Am Acad Dermatol.

[6] Birdi N, Laxer RM, Thorner P, Fritzler MJ, Silverman ED (1993). Localized scleroderma progressing to systemic disease. Case report and review of the literature. Arthritis Rheum.

[7] Venturi M, Pinna AL, Pilloni L, Atzori L, Ferreli C, Rongioletti F (2017). Bullous morphoea: a retrospective study. Clin Exp Dermatol.

[8] Garófalo Gómez N, Novoa López L, Gómez García AM, Méndez Méndez M (2012). Linear scleroderma en coup de sabre and epilepsy: presentation of a case in a child. Neurologia.

[9] Nguyen K, Atty C, Ree A (2020). Linear scleroderma en coup de sabre presenting with seizures. Radiol Case Rep.

[10] Amaral TN, Peres FA, Lapa AT, Marques-Neto JF, Appenzeller S (2013). Neurologic involvement in scleroderma: a systematic review. Semin Arthritis Rheum.

[11] Kister I, Inglese M, Laxer RM, Herbert J (2008). Neurologic manifestations of localized scleroderma: a case report and literature review. Neurology.

[12] Van der Veken D, De Haes P, Hauben E, Teughels W, Lambrechts P (2015). A rare cause of gingival recession: morphea with intra-oral involvement. Oral Surg Oral Med Oral Pathol Oral Radiol.

[13] Pace C, Ward SE, Pace A (2010). A rare case of frontal linear scleroderma (en coup de sabre) with intra-oral and dental involvement. Br Dent J.

[14] Tang MM, Bornstein MM, Irla N, Beltraminelli H, Lombardi T, Borradori L (2012). Oral mucosal morphea: a new variant. Dermatology.

[15] Taniguchi T, Asano Y, Tamaki Z (2014). Histological features of localized scleroderma 'en coup de sabre': a study of 16 cases. J Eur Acad Dermatol Venereol.

[16] Weibel L, Sampaio MC, Visentin MT, Howell KJ, Woo P, Harper JI (2006). Evaluation of methotrexate and corticosteroids for the treatment of localized scleroderma (morphoea) in children. Br J Dermatol.

[17] Uziel Y, Feldman BM, Krafchik BR, Yeung RS, Laxer RM (2000). Methotrexate and corticosteroid therapy for pediatric localized scleroderma. J Pediatr.

[18] Kroft EB, Groeneveld TJ, Seyger MM, de Jong EM (2009). Efficacy of topical tacrolimus 0.1% in active plaque morphea: randomized, double-blind, emollient-controlled pilot study. Am J Clin Dermatol.

[19] Seyger MM, van den Hoogen FH, de Boo T, de Jong EM (1998). Low-dose methotrexate in the treatment of widespread morphea. J Am Acad Dermatol.

[20] Kreuter A, Gambichler T, Breuckmann F (2005). Pulsed high-dose corticosteroids combined with low-dose methotrexate in severe localized scleroderma. Arch Dermatol.

